# Classification of variants of uncertain significance in *BRCA1* and *BRCA2* using personal and family history of cancer from individuals in a large hereditary cancer multigene panel testing cohort

**DOI:** 10.1038/s41436-019-0729-1

**Published:** 2019-12-19

**Authors:** Hongyan Li, Holly LaDuca, Tina Pesaran, Elizabeth C. Chao, Jill S. Dolinsky, Michael Parsons, Amanda B. Spurdle, Eric C. Polley, Hermela Shimelis, Steven N. Hart, Chunling Hu, Fergus J. Couch, David E. Goldgar

**Affiliations:** 1 Huntsman Cancer Institute, Salt Lake City, UT USA; 2 Ambry Genetics Laboratories, Aliso Viejo, CA USA; 3 Department of Pediatrics, University of California Irvine, Irvine, CA USA; 4 Molecular Cancer Epidemiology Lab, QIMR Berghofer Medical Research Institute, Brisbane, Australia; 5 Department of Health Sciences Research, Mayo Clinic, Rochester, MN USA; 6 Department of Laboratory Medicine and Pathology, Mayo Clinic, Rochester, MN USA; 7 Department of Dermatology, University of Utah School of Medicine, Salt Lake City, UT USA

**Keywords:** VUS, *BRCA1*, *BRCA2*, clinical history, likelihood ratio

## Abstract

**Purpose:**

Genetic testing of individuals often results in identification of genomic variants of unknown significance (VUS). Multiple lines of evidence are used to help determine the clinical significance of these variants.

**Methods:**

We analyzed ~138,000 individuals tested by multigene panel testing (MGPT). We used logistic regression to predict carrier status based on personal and family history of cancer. This was applied to 4644 tested individuals carrying 2383 *BRCA1/2* variants to calculate likelihood ratios informing pathogenicity for each. Heterogeneity tests were performed for specific classes of variants defined by in silico predictions.

**Results:**

Twenty-two variants labeled as VUS had odds of >10:1 in favor of pathogenicity. The heterogeneity analysis found that among variants in functional domains that were predicted to be benign by in silico tools, a significantly higher proportion of variants were estimated to be pathogenic than previously indicated; that missense variants outside of functional domains should be considered benign; and that variants predicted to create de novo donor sites were also largely benign.

**Conclusion:**

The evidence presented here supports the use of personal and family history from MGPT in the classification of VUS and will be integrated into ongoing efforts to provide large-scale multifactorial classification.

## INTRODUCTION

Genetic testing for germline pathogenic variants in *BRCA1* and *BRCA2* is widely used in clinical practice to identify individuals at increased risk of breast, ovarian, and other cancers. One factor limiting the clinical utility of such testing is the prevalence of rare sequence variants of uncertain clinical relevance in these genes. These are primarily missense changes but also include in-frame deletions and insertions, and variants (both intronic and exonic) that may affect splicing efficiency.^1–4^

Initial studies aimed at establishing the clinical relevance of variants of unknown significance (VUS) in *BRCA1* and *BRCA2* used clinical genetic testing results from ~60,000 individuals.^2^ Likelihood ratios for individual variants were derived using a logistic regression equation based on the characteristics of the personal and family histories of the individuals with known pathogenic variants (largely loss of function [LOF]) compared with individuals who did not have *BRCA1* or *BRCA2* pathogenic variants or VUS. In that analysis, it was estimated that, overall, 20% of the VUS analyzed were pathogenic; in subsequent analysis^3,5^ the proportion of variants with certain in silico characteristics based on the A-GVGD prediction model^3^ had estimated proportion of pathogenic variants of 0.81 (A-GVGD score C65) while other bioinformatics categories were predicted to contain a very small fraction of pathogenic variants. The values derived from theses analyses of in silico prediction are posted on the website^6^ and are used as prior probabilities in multifactorial models for classification of VUS by the BRCA1/2 expert panel (ENIGMA) and other groups.^7^

Because of the efforts of commercial labs, independent research efforts, and in particular the work of the ENIGMA consortium^8,9^ over the ten years following the initial study, many VUS have been reclassified as pathogenic or benign with respect to cancer risk. However, we note that due to the increased volume of genetic testing, the absolute numbers of individuals receiving an inconclusive *BRCA1/BRCA2* test result are still quite large, with roughly equal numbers of VUS (7360) and pathogenic/likely pathogenic variants (6968) in *BRCA1/BRCA2* listed in the National Institutes of Health (NIH) repository ClinVar (https://www.ncbi.nlm.nih.gov/clinvar; accessed 17 May 2019).^10^

Moreover, clinical characteristics of patients undergoing *BRCA1/BRCA2* testing have also changed over time. For example, *BRCA1/BRCA2* testing criteria have broadened and now include additional indications such as personal history of pancreatic cancer and unaffected women with only minimal family history. In addition, individuals with constellations of cancer beyond breast and ovarian frequently undergo *BRCA1/BRCA2* testing as part of multigene panel testing (MGPT).^11^

Considering these changes in genetic testing practices, we re-examined the utility of a clinical history–based approach for classification of *BRCA1/BRCA2* VUS in a cohort of >170,000 individuals undergoing hereditary cancer MGPT to develop prediction models that in turn allow inferences to be made about individual variants that can be included in multifactorial classification models. The results can then be used to provide updated calibration of in silico predictors that are often used as prior probabilities in these models.^7^

## MATERIALS AND METHODS

### Source of data and variants analyzed

The data analyzed in this report came from the large database of patients who underwent MGPT at Ambry Genetics (Aliso Viejo, CA) from March 2012 to December 2016. MGPT included comprehensive analysis of 5–49 genes, depending on the panel ordered.

Clinical history information was obtained from test requisition forms (available at https://www.ambrygen.com/file/material/view/984/Cancer_Comp_TRF_0918_final.pdf), and entered into Ambry’s database in a standardized manner by a team of trained clinical data curators. Ambry has previously shown that information reported on the TRF is accurate when compared with clinical records and pedigree drawings.^12^ This study has been exempted from review by the Western Institutional Review Board.

A total of 154,653 individuals were tested for *BRCA1* and *BRCA2*. Of these, 10,534 were excluded due to insufficient information regarding personal or family history of cancer, leaving 144,119 potential subjects. Lastly 5777 were excluded from the analysis because they had a pathogenic or likely pathogenic (VLP) variant in another gene (*ATM*, *PALB2*, *BARD1*, *CDH1*, *CHEK2*, *MLH1*, *MSH2*, *MSH6*, *NBN*, *NF1*, *PTEN*, *RAD51C*, *RAD51D*, *TP53*). After these exclusions, 138,342 individuals tested for *BRCA1* and *BRCA2* were eligible for analysis who had either a pathogenic variant, VLP or VUS in *BRCA1* or *BRCA2*, or were MGPT negative; that is, had no pathogenic (or VLP) variant identified in any of the other breast cancer susceptibility genes tested.

### BRCA prediction models

To construct a predictive model to be applied to VUS/VLP, a logistic regression model was used comparing the clinical histories (CH) of individuals with known pathogenic variants versus those with no reportable variant in these genes. We have included variants classified as VLP here as the analyses could in many cases generate evidence to move them into the pathogenic category. For the analysis of *BRCA1*, individuals who had pathogenic variants, VLP or VUS in *BRCA2* were excluded, leaving 131,352 included in the analysis of that gene. Conversely, probands with pathogenic variants, VLP, or VUS in *BRCA1* were excluded for the *BRCA2* analysis leaving 131,465 eligible. In total there were 14 parameters included for personal cancer history and 15 for family history, making 29 total clinical history parameters to be estimated in the logistic regressions. All logistic regressions included personal and family histories of the following cancers: breast cancer, ovarian cancer, pancreatic cancer, and prostate cancer. For breast cancer, age at diagnosis was categorized as <50 years or ≥50 years, and for ovarian and prostate cancers, ages were categorized as <60 years or ≥60 years. We also included ductal carcinoma in situ (DCIS), bilateral breast cancer, and male breast cancer as separate predictors. No age considerations were applied for pancreatic cancer. For personal history of breast cancer, additional subcategories were incorporated within each age group according to triple-negative (TN) status: TN, not TN (e.g., ER+), or status unknown. For family history, cancer counts were restricted to first- and second-degree relatives (maternal and paternal combined), with the number of affected relatives categorized as 0, 1, 2, 3+ for breast cancer and 0, 1, 2+ for the other cancer types.

Since index cases of different ethnic backgrounds might be expected to present with different distributions/frequencies of variants and different distributions of personal and family histories of cancer,^13–16^ we performed separate logistic regression analyses for each of four racial/ethnic groups: (1) Caucasian plus mixed or unknown race/ethnicity, (2) African American, (3) Asian, and (4) Hispanic. The predicted probabilities *r*_k_ of carrying a pathogenic variant for each tested individual were then derived using the predict option in Stata. We denote *r*_0_ to be the corresponding probability under the null hypothesis that the variant is unrelated to family history, or equivalently the prior probability of a pathogenic variant in the tested population. This is estimated by the overall proportion of individuals who have a pathogenic variant in the given gene (rather than a normal sequence). For example, using the data in Table 1, for the European group for *BRCA1*, *r*_0_ = 1706/(1706 + 108,602) = 0.015. We have shown previously that the required likelihood ratio (LR)

**Table 1:** Number of individuals and variants included in the study by gene and classification

Gene	Racial/ethnic group	Tested	Pathogenic	VLP	VUS	No variant
*BRCA1*^a^	European	95,607	1457	52	824	93,274
	Mixed/unknown	15,796	249	20	199	15,328
	African American	8545	193	10	166	8176
	Asian	5274	105	17	170	4982
	Hispanic	7693	189	10	95	7399
	**Total**	132,915	2193	109	1454	129,159
*BRCA2*^b^	European	96,641	1546	86	1735	93,274
	Mixed/unknown	16,025	281	17	399	15,328
	African American	8734	201	10	347	8176
	Asian	5371	118	4	267	4982
	Hispanic	7775	160	7	209	7399
	**Total**	134,546	2306	124	2957	129,159

*VLP* likely pathogenic variant, *VUS* variant of unknown significance.

^a^Excludes individuals with pathogenic or VUS/VLP variants in *BRCA2*.

^b^Excludes individuals with pathogenic or VUS/VLP variants in *BRCA1*.

$$\frac{{L[CH|V\;is\;pathogenic]}}{{L[CH|V\;is\;neutral]}}$$

is given by:

$$\frac{{r_k\left( {1 - r} \right)}}{{(1 - r_k)r_0}}$$

That is, the odds ratio of the predicted probability that an individual with the given family history is a carrier of a pathogenic variant against the corresponding probability under the null.^2^ For each variant, LRs were multiplied for each individual carrying that variant (potentially in different race/ethnicity groups) to arrive at a per-variant LR.

### Heterogeneity analysis

To estimate the proportion of pathogenic variants in the data set that are likely to be clinically significant as a function of bioinformatically predicted classifications, we performed a heterogeneity analysis analogous to that used previously in linkage analysis. Specifically, the required likelihood for a given class C is given by:

$$\mathop {\prod }\limits_{i = 1}^{N_C} [\alpha LR_i + (1 - \alpha )]$$

where *N*_*C*_ is the number of variants in the class and *LR*_*i*_ is the combined likelihood ratio for all probands carrying the *i*th variant.

This likelihood (in practice, the log-likelihood) is then maximized over α and approximate 95% confidence intervals (CIs) can be constructed by finding those values of α where 2ln(likelihood) differs by 3.84 from the −2ln(likelihood) at the maximum value. Hypotheses regarding differences in values of α as a function of partitions of the total variant space are performed using likelihood ratio tests.

## RESULTS

A total of 2383 distinct VUS/VLP in 4644 tested probands were identified through MGPT and were analyzed using the methods described above. Table 1 displays the *BRCA1* and *BRCA2* variant status of these individuals by racial/ethnic group. Tables 2 and 3 show the estimated odds ratios (OR) and corresponding 95% confidence intervals for the personal and family history factors, respectively, in the model as predictors of *BRCA1* and *BRCA2* pathogenic variant status across the four race/ethnicity groups. The numbers of tested individuals in each personal and family history category for each race/ethnicity group are provided in Supplementary Tables 1 and 2. For all racial and ethnic groups, the area under the receiver–operator curve (AUC) for the predicted pathogenic variant status were higher for *BRCA1* (0.79–0.83) than for *BRCA2* (0.66–0.70), due primarily to the higher predictive power of ovarian cancer and the association with triple-negative breast cancer in *BRCA1*. For *BRCA1*, the African American sample had the highest AUC (0.83), which was significantly higher than that for Caucasians. This is likely due to the higher prevalence of triple-negative breast cancer cases among African Americans. For *BRCA2*, the AUC was highest for the Asian sample (0.70) though not significantly different from the other racial/ethnic groups

**Table 2:** Odds ratios and corresponding 95% confidence intervals for personal history factors as predictors of *BRCA1* and *BRCA2* pathogenic variant carrier status

	*BRCA1*				*BRCA2*			
Phenotype	Caucasian	African American	Asian	Hispanic	Caucasian	African American	Asian	Hispanic
DCIS	1.0 (0.7, 1.3)	0.2 (0.0, 1.2)	0.8 (0.2, 2.6)	0.6 (0.2, 2.0)	1.3 (1.1, 1.6)	0.9 (0.4, 1.9)	0.5 (0.2, 1.2)	1.6 (0.9, 3.1)
TN + dx <50	15.9 (13.4, 18.9)	15.7 (9.8, 25.2)	23.5 (10.9, 50.6)	25.9 (15.7, 42.8)	3.1 (2.3, 4.0)	3.4 (1.9, 5.9)	1.5 (0.5, 4.3)	2.1 (0.9, 4.9)
TN + dx ≥50	5.1 (4.2, 6.3)	2.8 (1.4, 5.4)	9.0 (3.1, 26.6)	4.6 (2.2, 10.0)	2.9 (2.3, 3.7)	1.5 (0.7, 2.9)	1.9 (0.7, 5.6)	0.9 (0.3, 3.0)
TN – dx <50	1.7 (1.4, 2.1)	2.5 (1.5, 4.2)	3.1 (1.5, 6.1)	2.9 (1.7, 4.9)	2.5 (2.1, 2.9)	2.4 (1.5, 3.9)	1.0 (0.6, 1.8)	2.0 (1.2, 3.3)
TN – dx ≥50	0.5 (0.4, 0.7)	0.5 (0.2,1.5)	1.3 (0.5, 3.6)	0.7 (0.2, 2.0)	1.5 (1.2, 1.7)	1.4 (0.8, 2.5)	0.9 (0.4, 1.8)	1.8 (1.0, 3.2)
TN . dx <50	3.8 (3.2, 4.5)	5.4 (3.3, 9.0)	4.2 (1.9, 9.1)	4.8 (2.9, 8.1)	2.4 (2.0, 2.8)	3.2 (1.9, 5.2)	0.9 (0.4, 1.9)	1.8 (1.0, 3.2)
TN . dx ≥50	1.1 (0.9, 1.4)	1.2 (0.5, 3.1)	2.0 (0.6, 6.0)	–	1.4 (1.2, 1.8)	1.4 (0.7, 2.8)	1.2 (0.5, 2.9)	1.2 (0.5,2.9)
Bilateral BC	1.6 (1.3, 1.9)	1.8 (1.2, 2.8)	1.7 (0.8, 3.4)	2.2 (1.2, 3.9)	1.2 (1.0, 1.5)	2.0 (1.4, 3.1)	0.8 (0.3, 1.9)	1.1 (0.5, 2.2)
OV <60	10.7 (9.2, 12.4)	8.6 (4.7, 15.7)	17.2 (8.7, 34.0)	17.5 (10.6, 28.8)	4.0 (3.3, 4.8)	3.0 (1.5, 6.2)	1.4 (0.6, 3.3)	3.9 (2.1, 7.1)
OV ≥60	4.2 (3.4, 5.2)	1.1 (0.1, 7.9)	12.0 (4.9, 29.2)	14.1 (7.3, 27.4)	4.9 (4.1, 5.8)	4.3 (2.0, 9.4)	4.7 (2.2, 10.0)	2.6 (1.0, 6.8)
Pancreatic	1.3 (0.8, 2.3)	2.3 (0.5, 10.3)	–	–	3.9 (3.0, 5.2)	2.4 (0.8, 7.3)	5.1 (1.8, 14.7)	6.3 (2.4, 17.0)
Male BC	1.1 (0.4, 3.1)	–	–	–	6.3 (4.6, 8.6)	4.0 (1.6, 10.2)	1.8 (0.2, 15.6)	5.6 (1.2, 26.1)
Pr Ca<60	1.1 (0.3, 4.3)	–	–	–	1.4 (0.6, 3.2)	3.2 (0.4, 27.1)	–	10.3 (1.2, 87.6)
Pr Ca≥60	1.9 (0.7, 5.1)	–	–	–	2.4 (1.4, 4.1)	9.7 (1.8, 51.4)	5.1 (0.5, 58.9)	16.3 (3.2, 83.8)

*BC* breast cancer, *DCIS* ductal carcinoma in situ, *TN* triple negative.

.

**Table 3:** Odds ratios and corresponding 95% confidence intervals for family history predictors of *BRCA1* and *BRCA2* pathogenic variant carrier status

No. of 1st/2nd relatives	*BRCA1*				*BRCA2*			
With BC <50 (vs. 0)	Caucasian	African American	Asian	Hispanic	Caucasian	African American	Asian	Hispanic
1	2.2 (2.0, 2.4)	3.1 (2.2, 4.4)	2.8 (1.7, 4.5)	2.7 (1.9, 3.9)	1.5 (1.3, 1.7)	1.3 (0.9, 1.8)	1.3 (0.8, 2.0)	1.5 (1.0, 2.2)
2	3.7 (3.1, 4.4)	5.1 (3.2, 8.2)	3.0 (1.3, 7.2)	5.0 (2.9, 8.7)	2.0 (1.6, 2.4)	1.4 (0.8, 2.4)	1.5 (0.7, 3.5)	1.3 (0.7, 2.7)
3+	5.3 (4.0, 7.0)	12.0 (6.5, 22.1)	8.3 (2.2, 32.1)	9.1 (4.4, 18.9)	3.2 (2.4, 4.3)	1.8 (0.7, 4.5)	3.1 (0.7, 13.9)	3.4 (1.4, 8.2)
BC ≥50 (vs. 0)								
1	0.9 (0.8, 1.1)	0.7 (0.5, 1.0)	1.5 (0.9, 2.3)	1.1 (0.7, 1.5)	1.1 (0.9, 1.2)	0.8 (0.6, 1.2)	2.4 (1.6, 3.6)	1.3 (0.9, 1.9)
2	0.7 (0.6, 0.8)	0.5 (0.2, 1.0)	0.6 (0.2, 2.0)	0.7 (0.4, 1.4)	0.9 (0.8, 1.1)	0.8 (0.5, 1.3)	1.4 (0.6, 3.0)	0.9 (0.5, 1.7)
3+	0.7 (0.5, 1.0)	0.7 (0.2, 1.9)	2.0 (0.4, 8.8)	0.9 (0.3, 2.7)	0.7 (0.6, 1.0)	1.4 (0.7, 2.6)	3.0 (1.1, 7.8)	0.4 (0.1, 1.6)
OC <60 (vs. 0)								
1	3.2 (2.8, 3.7)	1.7 (1.0, 2.9)	3.4 (1.9, 6.2)	1.9 (1.2, 2.9)	1.4 (1.2, 1.7)	2.1 (1.3, 3.2)	1.5 (0.8, 2.8)	1.4 (0.8, 2.2)
2+	5.4 (4.1, 7.1)	0.4 (0.0, 2.9)	4.8 (1.2, 19.1)	3.0 (1.4, 6.6)	1.4 (0.9, 2.1)	0.6 (0.1, 4.2)	1.0 (0.1, 7.6)	1.0 (0.3, 3.4)
OC ≥60 (vs. 0)								
1	1.7 (1.4, 2.0)	2.3 (1.2, 4.1)	1.8 (0.8, 4.1)	1.9 (1.0, 3.6)	1.6 (1.3, 1.8)	0.7 (0.3, 1.5)	1.2 (0.6, 2.6)	1.5 (0.8, 2.9)
2+	1.6 (0.9, 2.8)	2.7 (0.6, 12.5)	5.6 (1.3, 25.0)	2.0 (0.3, 15.4)	1.9 (1.2, 3.0)	1.0 (0.1, 7.1)	–	1.6 (0.4, 7.0)
Pancreatic cancer								
1	1.1 (0.9, 1.3)	0.9 (0.5, 1.6)	0.7 (0.2, 1.9)	1.2 (0.7, 2.3)	1.3 (1.1, 1.5)	1.6 (1.0, 2.5)	1.7 (0.9, 3.1)	2.1 (1.3, 3.3)
2+	0.7 (0.4, 1.1)	2.0 (0.7, 5.5)	1.3 (0.2, 10.0)	2.6 (1.0, 6.6)	1.7 (1.3, 2.2)	1.1 (0.3, 3.6)	0.6 (0.1, 4.5)	0.4 (0.1, 2.9)
MBC	1.0 (0.7, 1.5)	0.9 (0.2, 3.7)	–	0.4 (0.0, 3.1)	2.5 (1.9, 3.1)	5.4 (3.0, 9.7)	–	3.1 (1.3, 7.5)
Prostate (vs. 0)								
1	0.9 (0.8, 1.1)	0.4 (0.2, 0.7)	0.9 (0.4, 1.9)	0.8 (0.5, 1.3)	1.3 (1.2, 1.5)	1.2 (0.8, 1.7)	1.8 (1.1, 3.0)	1.3 (0.8, 2.0)
2+	0.7 (0.5, 0.9)	0.5 (0.2, 1.1)	0.5 (0.1, 4.3)	0.4 (0.1, 1.4)	1.5 (1.3, 1.8)	0.9 (0.5, 1.6)	1.9 (0.6, 6.7)	2.1 (1.1, 3.9)
Total model AUC	0.79 (0.77–0.80)	0.83 (0.80–0.86)	0.79 (0.74–0.83)	0.82 (0.79–0.85)	0.67 (0.66–0.69)	0.68 (0.64–0.72)	0.70 (0.65–0.75)	0.66 (0.62–0.71)

*AUC* area under the curve, *BC* breast cancer, *MBC* male breast cancer, *OC* ovarian cancer.

### Variant classification

The calculated log-likelihood ratio scores and number of probands for each variant observed are shown in Supplementary Tables 3 (*BRCA1*) and 4 (*BRCA2*).

Assuming the previous prior probabilities based on the data in Tavtigian et al.,^3^ LRs from the present study provide evidence to support classification of 26 VUS with prior probabilities of pathogenicity between 0.29 and 0.81. Of these, 19 could be classified as benign or likely benign and 7 as pathogenic or likely pathogenic. In addition, LRs for 15 variants with an assumed prior probability of pathogenicity of 0.03 (variants in key functional domains that have A-GVGD scores of C0, indicative of neutrality) provide evidence to support a benign classification, as they were associated with odds of greater than 10:1 against pathogenicity. It should be emphasized here and in subsequent discussion that for many of these variants there is likely other evidence that is not considered in this paper; true clinical classifications should take into account all other available data, such as the multifactorial summary for a large number of variants in Parsons et al.^7^

### VUS with clinical history LRs indicative of high probability of pathogenicity

There were 22 variants labeled as VUS that had odds of >10:1 in favor of pathogenicity; of these, seven were missense variants not located in functional domains and with no bioinformatics evidence of interfering with normal splicing. Among the other 15, *BRCA1* EX16–18dup was notable with odds in favor of pathogenicity of 1895:1 and thus should be reclassified as pathogenic if considered appropriate in the context of other supporting evidence. Another variant, *BRCA1* c.5332G>A; p.D1778N, occurring at the last nucleotide of exon 21, had a moderate probability of damage to the wild-type splice donor, and odds in favor of pathogenicity of 884:1 based on clinical histories of eight individuals with this variant. If we assume the previously estimated prior probability of 0.34,^5^ this variant has a posterior probability of pathogenicity of 0.997 and can also be considered pathogenic based on multifactorial likelihood analysis, assuming no conflicting data from other sources. *BRCA2* c.383A>G, for which bioinformatic analysis indicates a moderate probability of creating a de novo splice donor with an assigned prior probability of 0.3 (http://priors.hci.utah.edu/PRIORS),^6^ had odds of 18:1 in favor of pathogenicity based on the clinical histories of three individuals with this variant.

### Analysis of variants classified as likely pathogenic (VLP)

We observed four variants (two in each gene) that were classified previously as likely pathogenic, but had odds of greater than 10:1 *against* pathogenicity in this analysis. In *BRCA1*, a frameshift variant, c.5578dupC, had odds against pathogenicity of ~11:1 based on personal and family histories of five index cases with this variant. This likely indicates that this variant results in a stable, almost full-length protein and does not undergo nonsense-mediated decay and that the truncated residues do not have any functional importance. In *BRCA2*, a splice variant c.517-2A>G had odds of 22:1 against pathogenicity based on six families. This variant was shown to result in deletion of *BRCA2* exon 7 and to lead to a frameshift.^17^ Lastly, analysis of *BRCA2* c.7878G>C;p.W2626C resulted in odds of 12:1 against pathogenicity based on 12 families. This variant was also evaluated in the Pruss et al.^18^ analysis and it was concluded that this was likely a hypomorphic allele, a finding consistent with our results.

### Heterogeneity analysis

Based on the family history log-likelihood ratios for each variant we estimated the proportion of variants in subgroups of variants that were pathogenic using the admixture model described in “Materials and Methods”. We first divided the missense variants into two groups based on their presence/absence in one of the known key functional domains: *BRCA1* nucleotides 1–294 and nucleotides 4987–5577 encompassing the start codon, the RING domain, and the BRCT repeats; and the DNA binding domain of *BRCA2* nucleotides 7669–9558. Then for those variants in one of these key domains we grouped them according to Align GVGD.^3^ Variants more likely to affect function by splicing than by an altered protein were categorized by their likelihood to create a de novo donor or damage the wild type as predicted by MaxENT scan.^5^ These groupings are reported on the website (http://priors.hci.utah.edu/PRIORS).^6^ Table 4 shows the results of these analyses for each of these subgroups. For each, we performed the analysis in two ways: first, including all variants that met the specified prior probability criterion irrespective of the Ambry Genetics classification (reflecting more the Easton et al.^2^ analysis); and then, removing all variants previously classified as pathogenic from these analyses. As shown in Table 4, 23% of VUS/VLP variants that were located in a key domain but were in A-GVGD class C0 were estimated to be pathogenic, compared with the previous estimate of 1% for this group. For variants within a key domain with an A-GVGD score of C65, for which with the prior probability was estimated to be 0.81 in the 2007 study,^2^ we found that the estimate to be numerically lower in the current analysis. The estimate was 0.77 when Ambry pathogenic variants (most classified after 2007, so would have been considered VUS in the original 2007 analysis) were included versus 0.60 when Ambry pathogenic variants were excluded. However, the newly estimated proportions were not significantly different (upper 95% confidence limits of 0.92 and 0.80, respectively).

**Table 4:** Heterogeneity analysis by bioinformatic groups

	Previous estimates^a^			This study: P+VLP+VUS			This study: VLP+VUS		
Bioinformatic group	NVAR	Estimate	95% CI	NVAR	Estimate	95% CI	NVAR	Estimate	95% CI
**Missense variants**	1177	0.12	0.08–0.17	2032	0.13	0.10–0.17	2007	0.09	0.06–0.13
Not in key domain	854	0.0	0.0–0.04	1507	0.019	0.0–0.05	1506	0.008	0.0–0.05
In key domain	323	0.35	0.26–0.45	525	0.44	0.35–0.53	501	0.36	0.26–0.44
C0	242	0.01	0.0–0.06	277	0.23	0.11–0.35	277	0.23	0.11–0.35
C15–C25	58	0.29	0.09–0.56	73	0.42	0.17–0.68	72	0.40	0.15–0.65
C35–C55	55	0.66	0.34–0.93	48	0.51	0.23–0.79	43	0.38	0.07–0.69
C65	98	0.81	0.61–0.95	127	0.76	0.59–0.92	109	0.59	0.28–0.90
**Potential splice variants**									
High damage to wild type	94	0.97	0.82–1.00	108	0.91	0.77–1.0	67	0.77	0.52–1.0
Moderate damage to wild type	66	0.34	0.15–0.55	49	0.79	0.54–1.0	40	0.61	0.27–0.95
Increased de novo donor	8	0.64	0.06–0.98	26	0.14	0.0–0.43	25	0.0	0.0–0.32
Moderate de novo donor	7	0.30	0.0–0.88	48	0.15	0.0–0.40	48	0.15	0.0–0.40
All de novo donor	15	NA	NA	74	0.14	0.0–0.33	73	0.08	0.0–0.26

*CI* confidence interval, *NVAR* number of variants, *P* pathogenic variant, *VLP* likely pathogenic variant, *VUS* variant of unknown significance.

^a^Easton et al.,^2^ Tavtigian et al.,^3^ Vallee et al.^5^

### Sensitivity analyses

We had included 16,500 subjects with unknown or mixed ethnicity within the large European/Caucasian group. To ensure that this did not bias our analyses, we performed a sensitivity analysis excluding patients with unknown/mixed ethnicity. All previously identified factors were significant in this analysis and ORs and model fits were similar (AUC 0.795 vs. 0.786).

In addition, we performed the analyses excluding individuals from the reference group who had been previously tested for *BRCA1/2* and found negative to avoid any biases with regard to family history for qualifying for previous *BRCA1/2* testing. Again, very little differences were observed in the parameter estimates and model performance (AUC 0.795 vs. 0.786).

## DISCUSSION

We have used a large clinical MGPT data set to inform classification for VUS/VLP in *BRCA1* and *BRCA2* based on analysis of personal and family history of >135,000 tested individuals. Of the 2383 such variants, 45 (5 of which were based on CH data from at least 5 probands) had LRs in favor of pathogenicity of >10.0 and 150 (57 of which had CH data from at least 5 probands) had odds against pathogenicity of >10.0. Integration of these LRs with in silico and other existing genetic and functional data should allow the clinical classification of significant numbers of VUS and VLP thus reducing uncertainty, improving the utility of genetic testing, and providing useful information to the individuals who carry these variants.

### Racial and ethnic differences in models

The large sample size of tested individuals here allowed us to fit models separately for each of four groups based on race/ethnicity. Tables 2 and 3 show some differences in strength of predictor variables between ethnic groups—for example, breast or ovarian cancer at older ages was a weaker predictor of *BRCA1* status in African American individuals than in the larger Caucasian set. Interestingly we observed that prostate cancer, particularly diagnosed over age 60, was a significant predictor of *BRCA2* pathogenic variant status in men, with relatively strong effects of both personal and family history of prostate cancer in Hispanics and African Americans. This could be of interest in setting testing criteria in these populations and may be due to a higher prevalence of aggressive prostate cancer in these populations and/or to higher Gleason scores in these populations that are known to be associated with germline *BRCA2* pathogenic variants.^19,20^

### Differences in model from the 2007 paper

The current study differs from the earlier similar study^2^ in several respects. Because testing criteria were stricter due to the cost of testing in 2007, the frequency of observed pathogenic variants was approximately double than that observed here due to ascertainment bias of the historic cohort; however, the larger sample size in the present study results in similar numbers of variants in the logistic regressions. Secondly, in the current study we were able to exclude individuals with pathogenic variants in several other genes from the non-*BRCA* group because of the current availability of multigene testing. Another important difference is that in the analyses presented here we included the triple-negative status of the breast cancer in the index case that is particularly important for *BRCA1* prediction as demonstrated in Table 2. Lastly we expanded our analysis to include two other cancers types, prostate and pancreatic, that were not included as predictors in the 2007 model. Although many of the factors related to breast and ovarian cancer were significant predictors of pathogenicity for both genes and across all races and ethnicities, there were a few differences, particularly with regard to pancreatic cancer and male breast cancer, which were shown to be important predictors for *BRCA2*, but not *BRCA1*. Interestingly, DCIS alone in the index case and having first-degree relatives with breast cancer over age 50 were predictors of the absence of a *BRCA1* pathogenic variant, but predictors of the presence of a *BRCA2* pathogenic variant.

### Heterogeneity analyses

The analyses of groups of variants by bioinformatics predictions shown in Table 4 illustrate a number of important findings. First, our analysis confirms previous indications that missense variants in regions of the gene that are not in functionally important domains of the protein are very unlikely to be pathogenic. In contrast to a previous study in which only 1% of variants predicted to be neutral (A-GVGD class C0) that were in recognized functional domains were estimated to be pathogenic, here we found that a significant fraction (23%, 95% CI 12–37%) of such variants were estimated to be pathogenic. Variants in this group with high odds of pathogenicity should be examined in detail, including detailed functional assays. For example, *BRCA1* c.5527G>C; p.Ala1843Pro, classified by Ambry as VLP, had odds of 21:1 in favor of pathogenicity (though based on only a single proband) and was classified as loss of function in both the assays of Woods et al.^21^ and Findlay et al.^22^ However in the in silico analysis, this variant was predicted to be neutral, with considerable variation observed in the multiple species sequence alignment with the variant amino acid proline observed. Lastly, the VLP variant *BRCA2* c.8188G>C;p.Ala2730Pro also scored as A-GVGD class C0 in the priors database but had odds of 18:1 in favor of pathogenicity based on five families; this variant displayed impaired homology directed repair (HDR) function in Hart et al.^23^

The estimates for the variants with predicted aberrant splicing as the more likely driver of pathogenicity indicated that for variants that were predicted to moderately damage the wild-type donor/acceptor site, nearly 80% could be predicted to be pathogenic, which was similar to that for those variants that were expected to severely damage the donor/acceptor. That only 80–90% of the variants that essentially impact the consensus splice site were as a group expected to be pathogenic is surprising, and may be due to the presence of alternative transcripts, in-frame exons that do not contain any important functional domains, both of which are known to be present in these genes (e.g*., BRCA1* c.594-2A>C^24^). Conversely, few if any of the variants predicted to create de novo donor sites were likely to be pathogenic, indicating that this is an unlikely mechanism of pathogenicity in these genes, or that the splice prediction algorithms currently do not use information on additional splice motifs or other context to differentiate which de novo donor sites are more likely to be used. It is also worth mentioning that the estimates in Vallee et al.^5^ were based on only eight variants and the confidence intervals were extremely wide; the present study had 25 variants in this category and the upper confidence limit was 0.32.

It would be of interest to compare our results here to other in silico predictors that have shown similar/higher correlations with pathogenicity. In addition, further discussion is needed regarding the calibration of the prior probabilities as delineated in the online database (http://priors.hci.utah.edu/PRIORS)^6^ as this is widely used and is integrated into a number of important resources such as the BRCA Exchange database (www.brcaexchange.org).^25^

### Caveats and limitations

At the individual level many variants (65%) were observed in only a single individual so that for these variants the results should not be overinterpreted. Only 177 variants (7.4%) were observed in 5 or more individual probands (Supplementary Tables 3 and 4). However, the general results for groups of variants should be sufficiently precise to draw valid conclusions, and to reclassify a substantial number of individual variants from VUS to the benign or likely benign categories, given other information.

### Conclusions

Based on the results presented here, it seems clear that personal and family history analyses in large clinical data sets are useful for providing statistical evidence about pathogenicity of VUS that then can be combined with other lines of evidence (e.g., cosegregation) in multifactorial models to derive clinically useful classifications for *BRCA1/2* variants.

The LRs calculated from this analysis can be utilized in a variant assessment scoring system such as the standards and guidelines recommended by the American College of Medical Genetics and Genomics (ACMG).^24–27^ For example our analysis suggests that a variant that is located outside of known functional domains, and that in silico predictions indicate is not likely to affect splicing, could be strong evidence (BS4) that the variant is benign based on the ACMG schema.^28^ LRs could also be more generally applied as “other data” to support pathogenic versus benign classification. For example, a variant with odds of >10:1 for or against pathogenicity based on five or more informative families could be considered as supporting evidence of pathogenicity or benign impact, respectively. Similar to cosegregation data, LRs could be used as even stronger evidence with increasing data such as additional families or increasing odds.

The results of this study bode well for applying this approach to other data sets from large clinical testing centers, and potentially from large population-based sequencing studies, provided family history data is sufficiently detailed. Moreover, this method should work for other cancer susceptibility genes for which the penetrance is high enough (e.g., *TP53, PALB2*) and/or there are rare cancers associated with pathogenic variants in the given gene that personal/family history is predictive of carrier status. Beyond the application to cancer susceptibility genes, the approach taken here should work when (1) phenotypic features are sufficiently predictive of individuals/families segregating a pathogenic variant in the gene of interest (e.g., AUC>0.65), (2) the sample size and frequency of pathogenic variants is sufficiently high that the number of index cases with pathogenic variants provides statistical power, and (3) relevant personal and family history data can be accurately and systematically collected.

The evidence presented in this paper should be integrated into ongoing efforts to provide large-scale multifactorial classification^7^ as well as translated directly into components of qualitative classifications such as the ACMG criteria used by many clinical testing laboratories,^26,28,29^ and further should be integrated into public resources displaying variant data (BRCA Challenge). These efforts will reduce the prevalence of VUS classifications that are so problematic from both provider and patient perspectives.

## Supplementary information

Supplementary Information

Supplementary Table3

Supplementary Table4
