# Supplementary material for: Classification of variants of uncertain significance in BRCA1 and BRCA2 using personal and family history of cancer from individuals in a large hereditary cancer multigene panel testing cohort
Source: Genet Med. 2019 Dec 19;22(4):701–8. doi: 10.1038/s41436-019-0729-1 (PMC7118020; doi:10.1038/s41436-019-0729-1)
Supplement: Supplementary file 1 — Supplementary Information [file 41436_2019_729_MOESM1_ESM.docx]

Supplementary Table 1. Number of tested probands in each phenotypic category used in the logistic regression.

|  | ***BRCA1*** | | | | | ***BRCA2*** | | | | |
| --- | --- | --- | --- | --- | --- | --- | --- | --- | --- | --- |
| **Phenotype** | **Combined** | **Caucasian** | **African Am** | **Asian** | **Hispanic** | **Combined** | **Caucasian** | **African Am** | **Asian** | **Hispanic** |
| Without BC | 55,782 | 47,817 | 2,711 | 1,761 | 3,274 | 55,783 | 47,798 | 2,803 | 1,797 | 3,339 |
| DCIS | 11,249 | 9,249 | 782 | 596 | 612 | 11,321 | 9,298 | 796 | 600 | 624 |
| TN+ dx <50 | 3,992 | 2,869 | 634 | 163 | 324 | 3,716 | 2,684 | 593 | 150 | 288 |
| TN+ dx ≥50 | 4,446 | 3,478 | 602 | 108 | 256 | 4,402 | 3,443 | 600 | 107 | 251 |
| TN – dx <50 | 19,866 | 15,802 | 1,473 | 1,244 | 1,388 | 20,046 | 15,957 | 1,493 | 1,246 | 1,343 |
| TN – dx ≥50 | 17,259 | 15,311 | 844 | 443 | 617 | 17,465 | 15,485 | 885 | 456 | 637 |
| TN . dx <50 | 10,709 | 8,663 | 776 | 486 | 765 | 10,619 | 8,594 | 780 | 486 | 757 |
| TN . dx ≥50 | 8,049 | 7,119 | 406 | 180 | - | 8,113 | 7,170 | 427 | 194 | 320 |
| Without Bi BC | 121,411 | 101,805 | 7,607 | 4,607 | 6,854 | 121,557 | 101,948 | 7,690 | 4,665 | 7,191 |
| Bilateral BC | 9,941 | 8.503 | 681 | 374 | 332 | 9,908 | 8,481 | 687 | 371 | 368 |
| Without OV | 121,245 | 101,508 | 7,902 | 4,553 | 6,706 | 121,497 | 101,725 | 7,992 | 4,621 | 7,097 |
| OV<60 | 5,556 | 4,704 | 232 | 297 | 316 | 5,354 | 4,545 | 224 | 282 | 301 |
| OV≥60 | 4.551 | 4.096 | 154 | 131 | 164 | 4,614 | 4,159 | 161 | 133 | 161 |
| Without Panc | 129,772 | 108,914 | 8,206 | 4,981 | 7,186 | 129,827 | 108,989 | 8,289 | 4,991 | 7,494 |
| Pancreatic | 1,580 | 1,394 | 82 | - | - | 1,638 | 1,440 | 88 | 45 | 65 |
| Male without BC and Female | 130,673 | 109,728 | 8,288 | 4981 | 7,186 | 130,728 | 109,800 | 8,313 | 5,010 | 7,541 |
| Male BC | 679 | 580 | - | - | - | 737 | 629 | 64 | 26 | 18 |
| Without Pr Ca | 130,663 | 109,669 | 8,288 | 4,981 | 7,186 | 130,752 | 109,773 | 8,347 | 5,031 | 7,540 |
| Pr Ca <60 | 322 | 295 | - | - | - | 328 | 299 | 12 | - | 11 |
| Pr Ca ≥60 | 367 | 344 | - | - | - | 385 | 357 | 18 | 5 | 8 |

Supplementary Table 2. Number of tested probands in each categoryof family history used in the logistic regression.

| **No. of 1^st^/2^nd^ relatives** | ***BRCA1*** | | | | | ***BRCA2*** | | | | |  |
| --- | --- | --- | --- | --- | --- | --- | --- | --- | --- | --- | --- |
| **with BC<50 (vs. 0)** | **Combined** | **Caucasian** | **Afr Am** | **Asian** | **Hispanic** | **Combined** | **Caucasian** | **Afr Am** | **Asian** | **Hispanic** |  |
| 0 | 75,183 | 75,183 | 5,303 | 3,692 | 4,859 | 89,734 | 75,400 | 5,414 | 3,745 | 5,130 |  |
| 1 | 27,391 | 27,391 | 2,190 | 1,041 | 1,751 | 32,443 | 27,357 | 2,190 | 1,046 | 1,836 |  |
| 2 | 6,282 | 6,282 | 615 | 211 | 440 | 7,510 | 6,236 | 603 | 209 | 457 |  |
| 3+ | 1,452 | 1,452 | 180 | 37 | 136 | 1,778 | 1,436 | 170 | 36 | 136 |  |
| BC>=50 (vs. 0) |  |  |  |  |  |  |  |  |  |  |  |
| 0 | 57,457 | 57,457 | 5,022 | 3,438 | 4,655 | 70,791 | 57,399 | 5,060 | 3,456 | 4,850 |  |
| 1 | 32,472 | 32,472 | 2,081 | 1,089 | 1,649 | 37,574 | 32,558 | 2,106 | 1,121 | 1,763 |  |
| 2 | 14,465 | 14,465 | 833 | 352 | 652 | 16,450 | 14,542 | 850 | 353 | 695 |  |
| 3+ | 5,914 | 5,914 | 352 | 102 | 230 | 6,650 | 5,930 | 361 | 106 | 251 |  |
| OC<60 (vs. 0) |  |  |  |  |  |  |  |  |  |  |  |
| 0 | 99,553 | 99,553 | 7,542 | 4,534 | 6,266 | 118,753 | 99,865 | 7,623 | 4,597 | 6,616 |  |
| 1 | 9,519 | 9,519 | 637 | 403 | 756 | 11,203 | 9,373 | 645 | 399 | 777 |  |
| 2+ | 1,236 | 1,236 | 109 | 44 | 164 | 1,509 | 1,191 | 109 | 40 | 166 |  |
| OC>=60 (vs. 0) |  |  |  |  |  |  |  |  |  |  |  |
| 0 | 101,264 | 101,264 | 7,771 | 4,686 | 6,739 | 121,115 | 101,365 | 7,864 | 4,768 | 7,083 |  |
| 1 | 8,182 | 8,182 | 460 | 266 | 401 | 9,344 | 8,196 | 456 | 268 | 421 |  |
| 2+ | 862 | 862 | 57 | 29 | 46 | 1,006 | 868 | 57 | - | 55 |  |
| Pancreatic Ca |  |  |  |  |  |  |  |  |  |  |  |
| 0 | 97,229 | 97,229 | 7,501 | 4,629 | 6,550 | 116,413 | 97,260 | 7,572 | 4,662 | 6,861 |  |
| 1 | 10,598 | 10,598 | 650 | 298 | 513 | 12,204 | 10,648 | 669 | 317 | 566 |  |
| 2+ | 2,481 | 2,481 | 137 | 54 | 123 | 2,848 | 2,521 | 136 | 57 | 132 |  |
| Female and Male without BC | 108,155 | 108,155 | 8,128 | 4,981 | 7,097 | 128,956 | 108,227 | 8,203 | 5,036 | 7,461 |  |
| MBC | 2,153 | 2,153 | 160 | - | 89 | 2,509 | 2,202 | 174 | - | 98 |  |
| Prostate (vs. 0) |  |  |  |  |  |  |  |  |  |  |  |
| 0 | 87,271 | 87,271 | 6,330 | 4,478 | 6,019 | 104,471 | 87,222 | 6,372 | 4,514 | 6,309 |  |
| 1 | 17,397 | 17,397 | 1,345 | 440 | 895 | 20,284 | 17,494 | 1,375 | 459 | 950 |  |
| 2+ | 5,640 | 5,640 | 613 | 63 | 272 | 6,710 | 5,713 | 630 | 63 | 300 |  |
